# Supplementary material for: Are carbon emissions trading and green financial instruments synergistic? -Comprehensive quantitative research based on content analysis
Source: PLoS One. 2024 Mar 7;19(3):e0298601. doi: 10.1371/journal.pone.0298601 (PMC10919601; doi:10.1371/journal.pone.0298601)
Supplement: S1 Data — (ZIP) [file pone.0298601.s001.zip › Data/地级市Z_F工作报告.zip!/║┌┴·╜¡/╣■╢√▒⌡╩╨/└┤╫╘╣1⁄2╓┌║┼í╛┬φ┐╦╩2╛▌═°í┐_2003.docx]

各位代表：

　　现在，我代表市人民政府向大会作工作报告，请予审议，并请市政协各位委员和列席会议的同志提出意见。

　　一、圆满完成上届人代会确定的各项任务

　　１９９８年以来，我们高举邓小平理论伟大旗帜，贯彻“三个代表”重要思想，按照市委确定的总体思路，自觉接受人大、政协的依法监督和民主监督，紧紧依靠全市人民，解放思想，奋发进取，圆满完成了市十一届人大一次会议确定的５年工作任务，开创了我市改革开放和现代化建设新局面。

　　国民经济在调整中持续快速健康发展。国内生产总值２００２年预计达到１２３２亿元，年均增长１１．２％，是历史上增长最快的时期之一。全市财政一般预算收入突破百亿元，地方财政一般预算收入年均增长１４．５％。抓住国家扩大内需的机遇，发挥国债项目的带动作用，积极扩大固定资产投资，５年累计完成１３００亿元，是前５年的２倍。哈飞微型车、哈药７－ＡＣＡ等１３９个项目竣工投入使用。大力推进结构调整，三次产业结构由２１．５：３２．２：４６．３调整为１６．２：３５．２：４８．６。工业结束了连续９年亏损局面，提前实现中央提出的改革与脱困目标。规模以上工业增加值年均增长１３．３％，汽车、医药、食品、电子信息４大优势产业增加值占规模以上工业增加值比重的５２％，年均增长１７．６％。农业总产值年均增长５．９％，畜牧业产值占农业总产值比重的４２．８％，比１９９７年提高１１．４个百分点。绿色食品发展迅速。粮经饲三元种植结构进一步优化。乡镇区街企业持续发展。产业化龙头企业发展到３９２户。农业基础设施建设加强，农业信息化和科技水平不断提高，绿化造林步伐加快。冰雪和生态旅游推动了全市旅游业的大发展，国内外游客年均增长１７．４％，旅游业总收入年均增长２２％。商品市场体系不断完善，商贸业态正在走向多样化、现代化。房地产、交通运输和其他服务业得到较快发展。

　　城市建设和管理水平显著提高。过去的５年是城市建设规模最大、速度最快的时期。市政基础设施建设总投资达３８５亿元，机场高速路、二环路、海城桥等重点路桥工程相继建成，新建、改造道路４５０万平方米。建成３２个城市住宅新区，农村住房砖瓦化率达到７０％。电力、通信、邮政、农村电网改造等取得历史性突破。开展城市环境综合整治，拆除各类占道房屋设施１６０万平方米，改造背街巷道１１００条，建设大型休闲、社区广场２３处，灯饰化景观街路２８条，新增绿地１６００公顷。马家沟综合整治取得显著成效，文昌污水处理厂形成３２．５万吨的一级日处理能力。完成城市供水应急工程。磨盘山水库工程已完成国家立项。在城市二环路以内推广电、气等清洁能源锅炉２７８０台，治理污染企业５２５家，拆除污染环境烟囱７３０座。实施“畅通工程”，交通管理达到国家二等优秀管理水平，城市公共交通事业取得长足发展。开通市政“１１０”等一批服务热线，城市管理综合执法试点取得较好效果。推进城市管理体制改革，“两级政府、三级管理、四级网络”管理模式逐步得到完善。经营城市迈出新步伐，采取市场化运作方式，推动了松北新区开发、城区企业搬迁和路桥等大型公共基础设施建设。建立了政府土地储备和挂牌交易制度。组织开展新一轮城市总体规划修编工作，完成建成区３５％的控详规划。启动了生态型园林城市建设。

　　经济体制改革不断深化。社会主义市场经济体制初步形成，市场在资源配置中的基础性作用得到初步发挥。国有企业改革取得进展，２４户重点优势国有企业实行产权多元化改革，１１０户国有大中型企业改组为公司制，改制面达到８２％；中小型企业改制１９８１户，改制面达到９０％。２３８户国有企业实施政策性破产或依法破产，４２亿元资产退出国有经济领域，核销呆坏账２９．１亿元。所有制结构发生较大变化，非公有制经济占国内生产总值的比重比１９９７年提高７．９个百分点。投融资体制改革取得明显成效。推行了部门预算、政府采购和“收支两条线”管理等财政改革。强化社会保障体系建设，“两个确保”、“三条保障线”得到较好落实。累计为２８．５万名国有企业下岗职工发放基本生活费、代缴社会保险费１４．９亿元，为３４．９万名离退休人员按时足额拨付养老金６７亿元。养老保险实现社会化发放，覆盖８７万人，覆盖率达到９６％。失业保险、医疗保险范围进一步扩大。３次提高最低生活保障标准，３０．７万人纳入低保范围。住房制度改革不断深化，金融、房地产、劳动力等各类要素市场发展较快。引进招商银行等金融机构在哈落户。地方金融机构为经济建设服务能力增强。农村完成第二轮土地承包，实行了税费改革。

　　对外开放水平逐步提高。累计完成进出口总值６１．３亿美元，批准外商投资项目５６０个。实施“走出去”战略，对外工程承包、劳务合作取得新进展。经济技术和高新技术产业开发区成为我市扩大对外开放重要基地和新的经济增长点。对俄经贸科技合作活跃，东北亚经贸科技合作区、哈尔滨铁路货运口岸、航空口岸作用日益增强。成功举办“第八届世界北方城市市长会议”。与国内各地区横向联合与协作取得新成果。

　　科技、教育及各项社会事业长足发展。科技创新步伐加快，建立哈工大、哈工程大学、哈尔滨农业３个国家级科技园和１６个高新技术企业孵化器，成立巨邦、亿阳等４个风险投资公司。锂离子电池、高压大功率变频调速装置等一批重大高新技术项目实现产业化，高新技术产业产值年均增长２３．１％，占工业总产值比重达到２５％，比１９９７年提高１２个百分点，增长速度居全国同级城市前列。民营科技企业实现技工贸总收入２００亿元，年均增长２７．７％。累计取得科技成果４８４０项，具有国际水平的占１９％，获国家专利４３２６项，连续两次评为全国科技进步先进城市。教育事业发展较快，１００％人口地区实现“两基”教育，农村教育综合改革取得成效，成人教育和地方高等教育资源优化重组成果显著，城乡教育对口支援和教育信息化建设居全国前列。城区９０％中小学校达到规范化学校标准。建立中高级人才市场，引进急需人才６０００余人，培养短缺人才１６０００余人。全民健身运动深入开展，竞技体育水平显著提高。“十冬会”筹办工作进展顺利。经国务院批准，获得冬奥会申办权。卫生改革不断深化，城乡居民健康状况得到改善。开通“哈尔滨数码城”，带动全社会信息化建设。统计、物价、审计、人防、司法、政策咨询、计划生育、爱国卫生、新闻出版、广播电视、民族宗教、台湾事务、武装、残联、气象、老龄、档案和地方志等方面工作取得新成绩。

　　人民生活总体达到小康水平。城市居民人均可支配收入达到７０００元，比１９９７年增长７０．５％；农村居民人均纯收入２７７５元，比１９９７年增长１１％。市区人均居住面积１０平方米，比１９９７年增长２８％；农民人均居住面积１８．６平方米，比１９９７年增长７．３％。居民消费结构发生显著变化，恩格尔系数明显下降，住房、教育、旅游、汽车、体育和文化渐成消费热点。注重关心贫困群众生活，开展扶贫济困等救助活动，下岗失业人员累计实现再就业２３．３万人，农村开发式扶贫取得明显成效。

　　社会主义精神文明和民主法制建设取得较大进步。城乡群众思想道德素质不断提高，崇尚科学、反对迷信的社会风气日渐形成。文化事业健康发展，累计获得国家级、省级各类文化艺术大奖２６８项。连续４次获得全国“双拥模范城”称号。社区建设得到强化，建成“星光老年之家”３００个。依法行政进一步加强，向市人大常委会提报地方性法规草案４３件，发布政府规章和规范性文件１５１件。办复省、市人大代表议案、建议２４１３件，办复省、市政协委员提案３８８７件。连续３年评为全省安全工作优秀达标城市。积极开展“严打”整治斗争，强化社会治安综合治理，依法打击“法轮功”邪教组织，维护了社会稳定。实行信访领导责任制，及时妥善处理大量群体事件和信访问题。依法打击制售假冒伪劣商品等违法行为，市场经济秩序进一步规范。推进政务公开、村务公开和厂务公开，实行服务承诺制、首问责任制。推进行政审批制度改革，行政审批事项减少５７．１％。基本完成政府机构改革，政府职能进一步转变。适应入世要求，建设学习型、调研型、决策型、服务型、网络型政府，公务员队伍素质不断提高。强化监督制约机制，促进了廉政建设。

　　世纪之交的５年，我们克服亚洲金融危机和国内有效需求不足的影响，战胜特大洪水等自然灾害，国民经济持续快速健康发展。特别是近两年，我们积极应对入世挑战，各项工作取得新成绩。工作中有这样几点体会：一是必须紧紧抓住“第一要务”，毫不动摇求发展。两个开发区管理机构合并、松北新区开发建设、沿江防洪工程等重大决策的实施，都说明一点，面对前进中的各种矛盾和问题，只有坚持发展是硬道理的思想和“三个有利于”标准，积极开拓，抢抓机遇，才能实现经济社会的不断进步。二是必须善于把握形势变化，创新思路谋发展。通过分层次推进工业经济战略调整，增强了工业整体竞争实力；绿色食品产业的开发，为农业和农村经济发展开辟了广阔空间；重大问题采用课题制，成功解决了冰雪大世界运作机制问题，探索了供热体制和冬季清冰雪机制改革的新途径。实践证明，思路决定出路，只有坚持与时俱进，不断创新，才能推动经济社会加速发展。三是必须突出重点，狠抓落实促发展。坚持抓大事、抓根本，重点工作实行专项推进，对照浙、闽等先进地区找差距，确立新目标，制定新措施，狠抓督促检查，保证了市委总体要求和部署的贯彻落实。四是必须坚持执政为民，凝聚人心保发展。视民情民意为“实事求是”最大的“事”，时刻把握人民群众的脉搏，注重关心、解决人民群众实际问题，维护了最广大人民群众的利益。几年来发展的实践启示我们，只要坚定不移地贯彻“三个代表”重要思想，紧紧抓住发展这一主题，始终与时代发展同步伐，与人民群众共命运，哈尔滨的未来就充满希望。

　　各位代表，我们取得的各项成绩，得益于党中央、国务院，省委、省政府和市委的正确领导，得益于人大代表、政协委员、民主党派、工商联和群众团体的监督和支持，得益于驻哈部队和武警官兵的鼎力相助，得益于港澳台同胞、海外侨胞、国内外友人的热情关注和真诚合作，凝聚了外来投资者、社会各界人士和全市人民的智慧和力量。在此，我谨代表市人民政府向全市人民和所有关心、支持、参与哈尔滨建设，为哈尔滨的发展、繁荣和文明做出贡献的同志们、朋友们，致以崇高的敬意和衷心的感谢！

　　在总结成绩的同时，我们也清醒地认识到前进中的矛盾和问题还很多。主要是结构和体制性矛盾仍然比较突出，特别是国有经济比重过大、产权制度改革不到位；民营经济总体规模不大，对经济增长拉动作用不强；社会保障体系还不健全，就业压力较大；城乡经济发展不够协调，县乡财政增长乏力；农村富余劳动力转移不畅，农民收入增长不快；对外开放程度不高，进出口额和利用外资规模较小；部分职能部门思想不够解放、服务意识不强，存在不正之风和腐败现象，经济发展环境还不够宽松。这些问题，必须在今后的工作中切实加以解决。

　　二、今后５年全面建设小康社会的战略目标和任务

　　未来５年，我们面临重要的战略机遇：党的十六大描绘了全面建设小康社会的宏伟蓝图，特别提出“支持东北地区等老工业基地加快调整和改造”、“加大对粮食主产区的扶持”，为我市加快发展创造了良好的政策环境；国家继续实施扩大内需政策，特别是调整和优化国债资金的使用方向，为我市加快工业结构调整和发展农村经济带来了新机遇。我们也面临前所未有的挑战：全国掀起新一轮改革发展浪潮，东部大发展，西部大开发，我市面临不进则退、慢进亦退的严峻形势。我们必须增强忧患意识，趋利避害，扬长避短，把握机遇，应对挑战。新一届政府将以邓小平理论和 “三个代表”重要思想为指导，全面贯彻党的十六大精神，按照市委“立足现有、挖掘潜能，追逐应有、创新发展”的总体要求，围绕富民强市这个中心，加快调整和优化经济结构，大力推进体制和科技创新，加速新型工业化进程，积极发展农业和农村经济，突出发展民营经济，努力扩大对外开放，完善社会保障体系，把哈尔滨建设成为国家机械制造业、高新技术产业、绿色食品、医药工业、对俄经贸科技合作基地和世界冰雪旅游名城，为实现全面建设小康社会的目标奠定坚实基础。

　　今后５年发展目标是：

　　——经济发展再上新台阶。在今后几年可预见的经济持续高增长期，力争“十五”后３年年均增长１２％。在确保完成“十五”计划的基础上，２００７年国内生产总值比２０００年翻一番，人均国内生产总值达到２５００美元。经济结构调整取得显著成效，经济、社会和环境协调发展。

　　——城乡面貌出现新变化。加快城市路桥等基础设施建设，形成“两轴四环十射”立体化交通格局。完成“一江一岛”升级改造和冰雪景观建设，城市特色更加突出。大气、水的环境质量达到相应功能区标准，城市污水处理率达到７０％，生活垃圾无害化处理率８５％，绿化覆盖率４０％，人均绿地面积９平方米，建成生态型园林城和环保模范城。加速小城镇建设，农村住房砖瓦化率达到７５％，城市化水平５０％。

　　——改革开放取得新进展。建立比较完善的社会主义市场经济体制，更加合理的所有制结构，更加有效的社会保障体系，更加规范、有序的市场环境和廉洁高效的行政管理体制。企业参与国际竞争能力增强，外贸进出口快速增长，利用外资水平大幅度提高，以发展对俄经贸科技合作为突破口，形成全方位、多层次、宽领域的对外开放格局。

　　——人民生活得到新改善。到２００７年，城市居民人均可支配收入超万元，农村居民人均纯收入３５００元，失业率控制在４．５％以内，恩格尔系数下降到３５％以下，人民生活更加富裕。加快磨盘山水库供水工程建设，形成日供水能力４５万立方米，让大多数城市居民吃上来自原始森林的洁净水。

　　——社会文明实现新进步。“以人为本”和“可持续发展”的理念深入人心，市民道德、文化素质和民主法制意识有较大提高。基本普及高中阶段教育，高等教育毛入学率达到８５％，农村“普九”成果进一步巩固。文化、卫生、体育等各项社会事业有较大发展。

　　为实现上述目标，实施６项战略。

　　一是科教兴市战略。依靠科技进步和提高劳动者素质，改善经济增长质量和效益。坚持自主技术创新，抢占科技制高点，集中力量抓好一大批技术起点高、规模效益好的高新技术项目，打造国际焊接城、新材料城和软件城。走新型工业化道路，用高新技术和先进适用技术改造传统产业，重振动力之乡、工具和亚麻之城的雄风，把汽车、医药、食品、电子信息培育成支柱产业。推进信息化，建设“数字哈尔滨”。落实教育优先发展战略方针，为经济和社会发展提供智力支撑。尊重知识、尊重人才、尊重创造，营造有利于人才干事业、干成事业的社会氛围。

　　二是改革推动战略。突出抓好国有企业和国有资产管理体制改革，探索公有制特别是国有制的多种有效实现形式，增强国有经济活力。毫不动摇地发展非公有制经济。深化农村经济体制改革，建设现代农业，增加农民收入，保护农民利益。深化投融资体制改革，确立企业投资主体地位，拓宽民间投融资渠道。适应入世新要求，加快行政管理体制改革和创新，完善政府经济调节、市场监管、社会管理、公共服务的职能，减少和规范行政审批。推进社会各领域改革，逐步消除各种体制性、机制性障碍，形成能干事的体制、干好事的机制，努力营造一切劳动、技术、知识、管理和资本的活力竞相迸发，一切创造社会财富的源泉充分涌流的社会环境。

　　三是开放牵动战略。坚持以开放促改革、促发展。积极参与全球经济分工和竞争，提高我市经济外向依存度。坚持“引进来”与“走出去”相结合，把利用外资与对外投资、扩大出口与优化进口结合起来，利用国际国内两个市场，优化资源配置，拓宽发展空间。通过体制和机制创新，实施“封闭式”管理，营造最佳政策环境，把开发区建成省、市扩大对外开放、拓展招商引资的龙头，优势产业高度聚集的新兴工业基地。围绕建设现代化新城区目标，完善松北新区规划，加大招商引资力度，加快基础设施和大项目建设，积极培育新的经济增长点，使松北新区成为我市经济发展的重要一翼。

　　四是可持续发展战略。控制人口规模，提高人口素质，优化人口结构；坚持资源合理开发和永续利用，形成低消耗、可循环利用的绿色生产、生活和消费方式；运用生态学原理和系统工程方法，持续实施“蓝天、碧水、绿地”工程，搞好城市绿化、环境治理和保护，全面改善城市生态环境，把哈尔滨建成适宜生活、创业、发展，充满生机、活力的新型城市。

　　五是城市化战略。加快区划调整，适度扩大中心城区规模，培育卫星城，发展小城镇，形成以市区为核心、中小城市和小城镇为支点，布局合理、功能互补、协调发展的现代化城镇体系。把农村工业化、农业产业化和发展乡镇企业与城市化紧密结合，调整农村生产力布局，促进乡镇企业、农业产业化龙头企业向小城镇集聚。加快城乡一体化进程，推动城市人才、科技、资本、教育、文化、卫生“六下乡”，实现城市和乡村之间、工业和农业之间的良性互动，扩大城乡就业，促进城乡经济协调发展。

　　六是申奥带动战略。动员全社会力量，办好“十冬会”，为申办冬奥会积累经验。不辜负国务院和全国人民重托，把申办冬奥会作为推动我市加速发展的历史机遇和长期任务，锲而不舍、百折不挠地申办下去。通过申办冬奥会，弘扬奥林匹克精神，提升市民综合素质，增强城市凝聚力，提高我市现代化水平，加快哈尔滨跻身国际化大都市行列的进程。

　　三、２００３年的主要任务

　　２００３年是深入贯彻十六大精神、全面建设小康社会的第一年，是新一届政府的开局之年，也是完成“十五”计划的关键一年，做好这一年的工作意义十分重大。我们要认真贯彻落实中央经济工作会议精神，按照省委常委扩大会议确定的６项原则，在市委的领导下，以与时俱进、开拓创新的思想观念，以奋发有为、敢于拼搏的精神状态，全面开创我市各项工作新局面。

　　全市经济和社会发展主要预期目标是：国内生产总值增长１１．５％，其中一、二、三产业分别增长６％、１２．５％、１２．５％。地方财政一般预算收入增长９％，全社会固定资产投资增长１５％，社会消费品零售总额增长１１．５％，外贸进出口总额增长１２％。城市居民人均可支配收入增长８％，农村居民人均纯收入增长５％。城镇登记失业率控制在４．３％以内，人口自然增长率控制在３．８‰以内。围绕上述目标，重点做好十方面工作。

　　（一）加大改革调整力度，走新型工业化道路

　　建立健全国有资产管理体制，打好深化国有企业产权制度改革攻坚战。建立代表国家履行出资人职责，享有所有者权益，权力、义务和责任相统一，管资产和管人、管事相结合的国有资产管理体制。改革国有资产管理机构，建立健全国有资产运营和监督管理体系，积极探索有效的经营体制和方式，实现国有资产保值增值。按照国有经济“有进有退”的原则，加快国有资产从一般竞争性领域退出步伐。深化国有企业改革，重点推进产权制度改革。除极少数必须由国家独资经营的企业外，绝大多数国有大中型企业都要积极推行股份制，建立完善的法人治理结构，实现投资主体多元化，促进混合所有制经济发展。强化服务，进一步放开搞活国有中小企业。

　　以企业信息化建设为切入点，提高工业经济整体素质。把信息化作为推进工业化进程的重要手段，由点到面，在企业全面推行信息化建设，将企业的体制创新、机制创新、科技创新和管理创新有机融合，促进企业实现根本性变革，增强核心竞争力。积极争取国家政策性破产，推进依法破产，加快劣势企业退出市场步伐，消除亏损源，降低亏损额和亏损面，改善工业经济效益结构。正确处理工业发展与环境保护和资源开发利用的关系，减少工业“三废”排放，降低生产综合能耗，发挥我市产业和劳动力资源优势，推进新型工业化进程。

　　着力培育优势产业、强势集团和知名品牌。积极争取国家支持老工业基地加快调整和改造的有关优惠政策，推进投资超亿元的重大技改项目，争取完成技改投资６０亿元以上，进一步提高汽车、医药、食品、电子信息４大优势产业的整体竞争力。集中力量培育强势企业集团，带动上下游企业纵向组合，延伸产业链条；推动同行业横向并购，扩大产业规模。以哈飞集团与巴西航空工业公司组建合资企业为契机，进一步扩大与国内外大公司特别是世界５００强企业联合。鼓励企业与绩优的上市公司重组做强，支持优势企业实施产品经营与资本经营双轮驱动，引导优质资产、优秀人才向大集团集中。做大做强哈啤、龙丹、完达山、金星等中国名牌，叫响哈药、哈电机、哈锅炉、哈汽轮机、哈飞、东安、哈麻双鹤、哈轴等品牌。

　　（二）以增加农民收入为中心，加快发展农业和农村经济

　　深化农业和农村经济结构战略性调整，全面提高农业竞争力。进一步优化粮经饲三元种植结构，依靠科技加快发展优质、绿色、特色农业，突出发展畜牧业。按照农业产业化、农村工业化和城镇化方向，大力发展农产品加工业、乡镇企业和非农产业。积极鼓励和引导农民发展一乡一业、一村一品特色经济。科学规划，加快县乡（镇）工业园区建设，带动县域经济快速发展。

　　推动龙头企业发展，进一步提高农业产业化经营水平。集中力量支持省、市重点龙头企业发展壮大，促进农业向二、三产业延伸。加快水稻、大豆、玉米、蔬菜、畜产品、山产品六大农业产业化基地建设。建立农业生产安全体系和农产品质量认证、检测体系，逐步与国际标准接轨。实施名牌带动战略，发展订单农业和外向型农业，拓展对外开放领域和层次。加强农村中介组织和经纪人队伍建设，提高农业组织化程度和农业综合效益。

　　加快小城镇建设，促进农村劳动力转移。加大小城镇建设投入，进一步完善现有小城镇载体功能，改革户籍管理制度，促进农民向城镇和非农产业转移，减少农民数量，增加农民收入。依托小城镇大力发展乡镇企业。加快农村县际县乡道路、农田水利、通信等公共基础设施建设，改善生产、生活条件。郊区农业要加快退出粮食生产，积极探索发展都市农业和率先实现农业现代化的路子。积极推动农村土地使用权有序流转和土地适度规模经营。继续推进农村税费改革，进一步减轻农民负担。

　　（三）以旅游和现代物流、会展等服务业为重点，大力发展第三产业

　　继续发展壮大旅游业。树立发展大旅游意识，合理调整旅游产业布局。在做大、做强、做精以冰雪大世界为主要内容的冰雪节和太阳岛等知名品牌基础上，开发四季旅游产品，推进野生动物园、四季冰雪大世界、异域风情和生态旅游示范区等项目建设。积极发展工业旅游亮点项目，进一步丰富旅游产品。利用冰雪节、区域旅游节活动和各类传播媒体，加大旅游产品促销力度。加快旅游企业重组步伐，提高行业竞争能力。整顿规范旅游市场，提高从业人员素质。旅游业总收入力争突破１００亿元。

　　大力发展现代物流业。加强物流基础设施的规划和建设，整合现有资源，发展第三方物流，培育骨干企业，推进企业物流社会化、现代物流产业化。加快建设哈尔滨温州“三马”国际商贸城、哈尔滨国际汽车城等一批大型专业批发市场，进一步完善粮食、蔬菜、畜产品等农产品市场体系，不断增强我市区域性物流中心功能。培育和发展新型商贸业态，推行代理制和连锁经营。加大国有商业企业改革力度，增强优势企业竞争力，加快劣势企业退出国有经济步伐。发展金融、保险、信息、咨询、法律、社区等服务业，引导居民扩大住房、汽车、旅游、通信、文化、体育等方面的消费，培育新的消费热点。

　　进一步发展会展经济。办好“哈洽会”、“冰雪节洽谈会”和各种产品展示、展销会，吸引人流、物流、信息流、资金流。

　　（四）加速发展和壮大民营经济，增强全市经济活力

　　积极创造民营经济发展政策环境。进一步解放思想，打破地区、行业、所有制界限，不限比例、不限速度、不限规模，放手、放胆、放量发展民营经济。鼓励民营企业依托市场自主创业，准许民营企业进入国家明令禁止以外的所有行业。降低创业门槛，放宽投资人资格、注册资本和投资方式的限制。以产业政策为标准，在税收、信贷、融资、项目审批、土地使用、技改贴息、进出口贸易等方面与国有企业一视同仁。区、县要以发展民营经济为突破口，增强经济实力和活力。

　　切实为民营企业搞好服务。建立健全以资金融通、信用担保、管理咨询、信息服务、法律服务、市场开拓和人才培训等为主要内容的民营企业服务体系。以开通市和区、县领导与民营企业大户“办事直通车”为重点，建立和完善政府部门与民营企业联系、协调、服务制度，悉心帮助民营企业解决生产经营中的困难和问题。对民营企业家在政治上关心、事业上支持，激发他们在哈尔滨创业和发展的热情。

　　大力培育民营企业生力军。鼓励、支持并积极引导民营企业以承包、租赁、联合、兼并、参股、控股、托管、收购等形式参与国有企业产权制度改革，促进民营企业快速扩张。帮助有条件的民营企业上市，支持民营企业开展自营进出口业务，进军国际市场。着力扶持民营科技企业，推进“小巨人”发展计划，进一步壮大光宇、九洲、圣泰、华崴、四海等一批具有巨大发展潜力的民营科技企业，打造全市经济发展新优势。

　　（五）大力优化经济发展环境，积极发展外向型经济

　　改善投资环境，降低社会成本。进一步实行政务公开，精简行政审批事项，简化审批程序，规范审批行为，减少收费项目和额度。创建市政府行政审批服务中心，一个窗口对外，一站式办理，一条龙服务，提高办事效率。进一步整顿和规范市场经济秩序，创造健康有序的市场环境。实施“信用哈尔滨”工程，加快城市信用体系建设步伐。加强政府职能部门队伍建设，强化制度约束，发挥社会监督作用，依法整治“勒卡索要”等不正之风。认真解决投资者投诉问题，切实维护其合法权益，营造亲商、安商、富商的社会氛围。

　　举全市之力大招商、招大商。建立健全目标责任制，充分调动各方面积极性，形成全市上下齐心协力抓招商引资的工作局面。组建招商公司，推行委托招商、产业招商和项目招商，借助各地驻哈商会、在哈企业，实行以商招商。积极采取各种方式利用外资。充分发挥开发区和松北新区的载体作用，再造体制、环境新优势，努力实现招商引资新飞跃。

　　全力构筑对外经贸新格局。积极推动贸易主体、出口市场、出口商品和贸易方式多元化，大力开拓国际市场。发挥优势，努力增加机电、高新技术产品和绿色食品出口。积极发展国际工程承包和劳务合作。进一步加强口岸建设，扩大口岸开放。加强同俄罗斯在医药工程、航天技术、新材料、焊接等领域的全面合作，构筑国家对俄经贸科技合作战略平台。增强城市辐射和集聚功能，推动省内外横向联合与协作。

　　（六）强化城市载体功能，塑造现代城市形象

　　全面实施生态型园林城市建设。树立“环境兴市”理念，建设城市生态功能区，启动四环路、铁路沿线等绿化廊道工程，辟建野生动物园、黄河公园，建设一批绿化区和绿化广场。科学实施“大树进城”工程，加快街路绿化和社区绿化。加强环境保护，继续整治马家沟，启动何家沟治理和向阳垃圾无害化处理厂工程，推进道里、南岗、松北新区等集中供热和区域连片供热工程，积极推广和采用新型能源供热，抓好城区原煤散烧和扬尘的控制及治理，推行工业企业清洁生产，全面实施污染物排放总量控制。

　　加快城市规划和重点工程建设。完成到２００７年全市近期建设规划、松北新区总体规划、重点地段控详规划和群力新区基础设施建设规划等４１项专项规划的编制工作。开工建设磨盘山水库供水工程，加快轨道交通一期工程前期工作，拓宽改造工农大街、道外十四道街、工电路等一批主干街路，打通拓宽一批“断头路”、“卡脖路”，进一步改造整修三、四类街路，完善城市路网系统。推进松花江城区段南岸改造工程，实施前进堤改线二期工程，配合省搞好２０２国道拓宽、四环路西段和第二松花江公路大桥、会展体育中心等工程建设。

　　继续加强城市管理和环境综合整治工作。坚持依法管理城市，全面开展城市管理相对集中处罚权工作。注重发挥区、街和社区组织的作用，完善城市管理体制。搞好环境综合整治，创建一批达标景观庭院、示范社区及社区广场。实施亮化、美化工程，整治重点区域、重要出城口、重点保护建筑和街坊的周边环境。把“市容”与“繁荣”紧密结合起来，在合理规划、搞好管理的前提下，积极开辟便民、利民街区。

　　进一步搞好经营城市工作。打造城市品牌，促进城市升值。深化投融资体制改革，拓宽基础设施项目融资渠道。组建城市建设投资公司，加强城市基础设施大项目建设筹融资工作。积极转让市政基础设施经营权，盘活城市资产。加快城市基础设施建设市场化运作步伐，招商建设污水和垃圾处理等项目。全面推行土地收购储备和招标拍卖制度，充分挖掘和发挥土地资产效益。

　　（七）加快各领域改革步伐，推进体制创新

　　深化行政管理、分配制度、公共事业等各项改革。坚持政企分开，精简、统一、效能和依法行政的原则，以转变政府职能为关键，继续深化行政管理体制改革，搞好事业单位改革。依据劳动、资本、技术和管理等生产要素按贡献参与分配的原则，推动分配制度改革。推进财政改革，全面实行部门预算、扩大国库集中收付和政府采购，建立公共财政体制框架。深化供热体制改革，加快实行住房分配货币化。推动机关后勤、公共交通、市政设施管护、供水、供气、物业等市场化改革。推进医疗机构和药品流通体制改革。

　　加快现代市场体系建设。完善商品市场体系，培育和发展产权、土地、劳动力和技术等要素市场。深化金融体制改革，搞好市商业银行资产重组和内部机制改革，支持其加快发展壮大。努力吸引更多的股份制商业银行和外资银行在我市设立分支机构。逐步建立统一的企业信用等级评价体系，增强全社会信用意识。

　　建立和完善社会保障体系。做好“两个确保”、“三条保障线”衔接工作，发展社会福利事业，推动“送温暖”、“一帮一”等活动经常化、制度化，不断改善贫困群众生活。深化城镇职工养老保险、失业保险、基本医疗保险制度改革。扩大社会保险覆盖面，搞好社会保险费征缴。通过调整财政支出结构、变现部分国有资产等形式，充实社会保险基金。落实扶持政策，改善就业环境，千方百计扩大就业和再就业。

　　（八）全面搞好各项社会事业，促进两个文明协调发展

　　加快科技创新步伐。健全科技进步目标考核体系，推进专利实施工程，重点突破影响和制约经济社会发展的关键领域和重点技术。完善技术创新体系，大力发展技术评估、技术市场等中介组织，加速科技成果的商品化、产业化。建立多元化投资机制，拓宽国内外风险投资渠道，积极为高速增长、相对成熟的高新技术企业上市搞好服务。加快电子政务建设，推进政府网上审批和网上在线服务。在城市建设、社会保障、社会治安等领域广泛应用信息技术，实施“数字哈尔滨”示范工程。

　　落实教育优先发展战略地位。增加教育投入，积极鼓励多渠道、多形式的社会集资和民间办学，继续办好市属高等院校。坚持义务教育均衡发展原则，巩固和扩大“两基”教育成果。推进课程改革，提高教育质量。扩大优质高中教育资源，实现城区普及高中教育目标。调整职业教育结构，促进职业中学、中专学校、技工学校的协作发展，为社会培养适用人才。发挥人才作用，稳定人才队伍。构筑人才市场体系，促进人才资源的市场化配置。

　　协调发展各项社会事业。挖掘我市历史文化资源，丰富历史文化名城内涵，发展各类文化事业和文化产业。优化卫生资源配置，加强农村卫生工作，完善卫生服务体系。稳定低生育水平，提高人口素质。推动群众性体育健身活动，提高竞技体育水平。办好“十冬会”和冰雪节，继续开展申办冬奥会工作。巩固“双拥模范城”成果。抓好外事侨务、民族宗教、台湾事务、新闻出版、广播电视、残联、老龄、武装、人防、档案和地方志等工作。

　　强化安全和稳定工作。切实落实生产、消防、交通等安全责任制，努力消除各类事故隐患，防止重特大事故发生。加强社会治安综合治理，依法打击各种刑事、经济犯罪和“法轮功”等邪教组织非法活动。继续开展隐蔽战线斗争，抓好“反恐”工作。加强群众来信来访工作，维护社会稳定。

　　加强社会主义精神文明建设。落实《公民道德建设实施纲要》，加强社会公德、职业道德和家庭美德教育。宣传和弘扬爱岗敬业、助人为乐、扶贫济困等典型。广泛开展文明城市、文明村镇、文明行业、文明社区和军警民共建等活动，在全社会营造“爱国守法、明礼诚信、团结友善、勤俭自强、敬业奉献”的道德风尚。

　　（九）解决热点难点问题，不断提高人民群众生活质量

　　抓住与人民群众切身利益息息相关的实际问题，重点办好１０件实事：安置下岗职工和失业人员８万人。对５０条段市政土路进行硬铺装。开辟、调整８条公交线路，更新公交车辆３００台。拆除危棚房１００万平方米，改善２万户居民居住条件。对全市２１７家物业管理企业进行清理、整顿，组建１５０个业主委员会，提高物业服务质量。启动建设５００公里县乡高等级路面工程。解决革命老区３００个村（屯）群众饮水难问题。建设１０个社区卫生服务中心，１５个社区卫生服务站。完成文昌污水处理厂二期工程，启动沿江９个排污口截流工程，拔除大烟囱２００座。全面启动太阳岛综合整治改造工程。

　　（十）加强政治文明建设，不断改进政府工作

　　发扬民主，依法行政。自觉接受人民代表大会及其常委会的监督，完善工作报告制度，认真执行人大及其常委会的决议、决定。主动加强与人民政协的联系，积极支持其履行政治协商、民主监督和参政议政职能。支持工、青、妇等群团组织开展工作。发挥政策咨询机构作用，完善了解民情、反映民意、集中民智、珍惜民力的决策机制，推进决策科学化和民主化。努力提高办理人民代表议案、建议和政协委员提案的质量。严格遵守宪法、法律和法规规章，切实做到依法决策、依法管理、依法办事。做好政府立法工作，完善行政执法责任制，加强行政复议工作。

　　改进作风，从严治政。牢固树立群众观点和实践观点，加强调查研究，深入基层、深入实际、深入群众，倾听群众呼声，关心群众疾苦，做到权为民所用，情为民所系，利为民所谋，把人民群众的利益维护好、实现好、发展好。大力倡导说实话、办实事、鼓实劲、求实效的工作作风，反对形式主义、官僚主义、虚报浮夸、滥用民力和办事拖拉的恶劣作风。加强廉政建设，坚决查处以权谋私案件，严惩腐败分子。

　　加强学习，提高素质。深入学习贯彻十六大精神，认真研究社会主义市场经济发展的客观规律，增强按照国际通行规则办事的能力，不断提高领导水平和执政能力。强化改革创新意识，尊重群众的首创精神，创造性地开展各项工作。居安思危，艰苦奋斗，始终保持奋发进取、昂扬向上的精神状态，永不自满、永不懈怠。增强公仆意识，爱岗敬业、勤政为民，聚精会神搞建设，一心一意谋发展。公正诚信，取信于民，以自身形象和行动，带动和影响全社会形成生机勃发、勤奋进取的良好风尚。

　　各位代表，我们已经站在新的历史起点。让我们继续高举邓小平理论伟大旗帜，全面贯彻“三个代表”重要思想和党的十六大精神，在市委的领导下，坚定信心，奋力开拓，夺取全面建设小康社会的新胜利。
